# Inconsistent Association between Perceived Air Quality and Self-Reported Respiratory Symptoms: A Pilot Study and Implications for Environmental Health Studies

**DOI:** 10.3390/ijerph20021491

**Published:** 2023-01-13

**Authors:** Yang Liu, Mei-Po Kwan, Zihan Kan

**Affiliations:** 1 Department of Geography and Resource Management, The Chinese University of Hong Kong, Hong Kong SAR, China; 2 Institute of Space and Earth Information Science, The Chinese University of Hong Kong, Hong Kong SAR, China

**Keywords:** perceived air quality, air pollution, self-reported respiratory symptoms, environmental health, socio-demographic difference, geographic context

## Abstract

As public awareness of air quality issues becomes heightened, people’s perception of air quality is drawing increasing academic interest. However, data about people’s perceived environment need scrutiny before being used in environmental health studies. In this research, we examine the associations between people’s perceptions of air quality and their self-reported respiratory health symptoms. Spearman rank correlation coefficients were estimated and the associations were tested at the 95% confidence level. Using data collected from participants in two representative communities in Hong Kong, the results indicate a weak but significant association between people’s perceived air quality and their self-reported frequency of respiratory symptoms. However, there are disparities in such an association between different genders, age groups, household income levels, education levels, marital statuses, and geographic contexts. The most striking disparities are between genders and geographic contexts. Multiple significant associations were observed for male participants (correlation coefficients: 0.169~0.205, *p*-values: 0.021~0.049), while none was observed for female participants. Besides, multiple significant associations were observed in the old town (correlation coefficients: 0.164~0.270, *p*-values: 0.003~0.048), while none was observed in the new town. The results have significant implications for environmental health research using social media data, whose reliability depends on the association between people’s perceived or actual environments and their health outcomes. Since inconsistent associations exist between different groups of people, researchers need to scrutinize social media data before using them in health studies.

## 1. Introduction

Poor air quality and its adverse health impacts have been a long-lasting public concern, especially in urban areas. Studies have shown that air pollutants such as volatile organic compounds (VOCs) [1], secondary inorganic aerosols (SIAs, e.g., ${\mathrm{SO}}_{4}^{2-}$, ${\mathrm{NO}}_{3}^{-}$, and ${\mathrm{NH}}_{4}^{+}$) [2], and fine particulate matter (e.g., PM_2.5_) [3] have either direct or indirect negative impacts on human physical and mental health [4,5,6,7]. Along with more studies, accumulation of scientific evidence, and propagation through social media, public awareness of poor air quality has heightened [8,9,10]. Correspondingly, people’s perception of air quality is drawing increasing academic interest in recent years.

The association between people’s perceived air quality and actual ambient air quality has been supported by specific visual and physiological pathways and epidemiological investigations. Two mechanisms of the human body’s reactions to air pollutants enable people to perceive the poor quality of ambient air. The first one is the visual effects of particulate matter. Particulate matter is not directly visible, but it can decrease the transparency of ambient air through non-selective scattering, which results in an outdoor haze [11] and an indoor halo [12]. The other mechanism is the irritation of the human respiratory system by irritant gases such as ozone. People then have more frequent and more severe symptoms in the respiratory system, e.g., cough, phlegm, and shortness of breath, when the ambient air is polluted [13]. Besides these visual and physiological pathways, studies have also shown that people’s perception of air quality is consistent with their actual ambient air quality and health outcomes through epidemiological investigations [14]. For example, Pantavou et al. [15] found that pedestrians’ perception of air quality is significantly related to their exposure to air pollutants. Zakaria et al. [16] have shown significant associations between traffic-related air pollutants and perceived air quality as well as between traffic-related air pollutants and respiratory symptoms. Shi et al. [17] found that perceived worse outdoor air quality has a significant and negative association with life satisfaction.

Given these consistent associations between people’s perceived air quality and the actual quality of ambient air, the effectiveness of people’s perception of air quality indeed validates the utility of social media data in environmental health studies. Conventional environmental health studies on air quality use data from monitoring stations [18], field surveys [19], and portable personal monitors [20], which are spatially or temporally sparse. Social media data (e.g., data extracted from Sina Weibo and Twitter) are recently drawing increasing attention since they have several advantages, including broad spatial coverage and high spatiotemporal resolutions [21], high flexibility in covering various health concerns [22], fast response to extreme events without presetting [23], and no requirement for extra devices. The most fundamental assumption underlying the validity of using these social media data is that people’s perceptions and linguistic expressions of air quality can accurately represent their ambient air quality as well as their health burdens from poor air quality. Consistent and significant associations between people’s perceptions and linguistic expressions of air quality from social media and their actual exposure to poor air quality have been examined and reported in many studies [21,22,24].

People’s perceptions of air quality should, however, be carefully examined in relation to various axes of socio-demographic differences and different geographic contexts. Several studies have shown that disparity in perceived air quality exists between different groups of people. For example, Guo et al. [25] found that people aged 40 or older, with a higher education level, and children with poor health conditions have much more negative perceptions of air quality. Ban et al. [26] and Cisneros et al. [27] both reported a difference between men and women in the awareness and risk perception of worse air quality. King [28] found that ethnic minorities such as Hispanics and blacks tend to report higher levels of air pollution. Most of these studies to date have paid attention only to the disparities in people’s perceived air quality, and few studies have examined the disparities in the association between people’s perceived air quality and their health outcomes. This is a very important disparity that may undermine the effectiveness of perception-based datasets such as social media data but has long been ignored. People’s perception of air quality may not be consistently associated with their health outcomes for different groups of people since their vulnerabilities to poor air quality may vary. For example, young adults have the strongest resilience to poor air quality while older adults and those with poor health conditions may have a much weaker resilience to poor air quality [29]. Correspondingly, similar reports of poor air quality may simply be complaints for young adults but may be critical indicators of adverse living conditions for older adults and those with poor health conditions. It is thus essential to examine whether disparities in the association between perceived air quality and health outcomes exist between different social groups.

To fill this research gap, this study aims at examining the association between people’s perceived air quality and their self-reported health outcomes with respect to different genders, age groups, household income levels, education levels, marital statuses, and geographic contexts. The perception of air quality and the health impacts of poor air quality on the human respiratory system in two communities in Hong Kong are chosen as a case study.

## 2. Materials and Methods

### 2.1. Survey Area and Study Population

**Figure 1:**
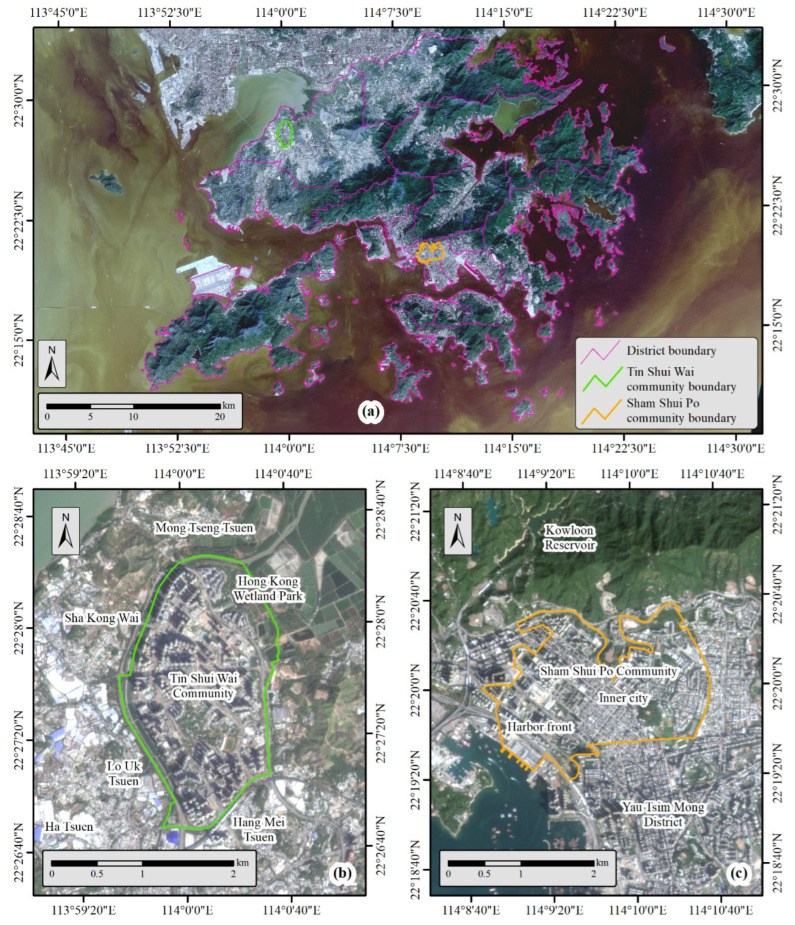
The geographic settings of SSP and TSW. (**a**). The locations of SSP and TSW in Hong Kong, (**b**). the geographic settings of TSW (new town), and (**c**). the geographic settings of SSP (old town).

The study area for this research is Hong Kong, which is one of the most densely populated cities in the world. It has a population of about 7.6 million and a land area of over 1100 square kilometers [30]. Hong Kong comprises Hong Kong Island, Kowloon Peninsula, and the New Territories with 18 districts. In this study, we selected two representative communities in Hong Kong to conduct the field survey. The first one is the Sham Shui Po (SSP) community and the other is the Tin Shui Wai (TSW) community (Figure 1).

Communities in Hong Kong are mainly old towns developed in earlier times and new towns developed since the 1950s. Old towns and new towns represent two geographic contexts with considerable differences in building density, urban planning, traffic networks, etc. [31]. SSP is chosen to represent the old towns in Hong Kong. It is a densely populated community, and the blocks included in this study have a total area of 5.35 km^2^, with a residential population of about 300,000 in 2018. On the other hand, TSW is chosen to represent the new towns in Hong Kong. It has a total area of 4.32 km^2^ and its residential population was also about 300,000 in 2018. SSP and TSW can well represent the geographic contexts of old towns and new towns in Hong Kong (Figure 1b,c). SSP belongs to the downtown area of Hong Kong, which is among the earliest-developed urban areas and is well-urbanized [32]. The major part of SSP, namely the inner city, has mostly lower and crowded buildings that are directly adjacent to numerous narrow roads, making air exchange less efficient. TSW is a town newly developed in the 1980s in northwestern Hong Kong [32], and is adjacent to the Hong Kong Wetland Park and Hong Kong’s fish farm regions. The social facilities and infrastructures in TSW are well designed with better open space and the roads are located far from residential buildings with green spaces between them as barriers. The entire TSW community is enclosed by villages and natural parks.

### 2.2. Questionnaire Design and Data Collection

In this study, people’s perceived poor air quality and its pressure on the human respiratory system are employed as a case study for examining the associations between them in different socio-demographic groups and geographic contexts. Note that information on people’s socio-demographic characteristics and geographic contexts is very difficult to collect from social media and thus we used a comprehensive questionnaire instead. The data were collected through questionnaires and face-to-face surveys, while the inconsistent associations are further discussed and generalized for commonly used social media data collection.

We used a questionnaire to collect data on people’s perceived air quality and their self-reported frequency of respiratory symptoms. On one hand, participants were asked three questions about their perception of air quality. These questions include (1) how severe they think that the air pollution was in their residential neighborhood in the last six months (hereafter named neighborhood air quality), (2) how severe they think that the air pollution was in the places they visit every week (hereafter named place air quality), and (3) how bad the overall air quality was in Hong Kong in the last six months according to their experience (hereafter named Hong Kong air quality). The data collected from these three questions are ordinal answers (scores) recorded with a 6-point scale, from no air pollution to severe air pollution for questions (1) and (2), and from very good air quality to very terrible air quality for question (3), respectively. A higher score means worse perceived air quality when compared to a lower score. On the other hand, the participants were asked three questions about their respiratory symptoms. These questions included how frequently they had the symptoms of (1) cough, (2) phlegm, and (3) shortness of breath in the last year. The data collected from these three questions are also recorded with a 6-point scale, from never to always, respectively. A higher score means a higher frequency of respiratory symptoms when compared to a lower score. Further, people’s chronic health conditions may affect their perceptions of air quality. Thus, we also required participants to report their chronic health conditions, including respiratory and other chronic health conditions.

The survey was carried out from 21 March 2021, to 12 September 2021, in a face-to-face manner. Participants were recruited from SSP and TSW via a stratified sampling process. The sample was stratified according to the proportions of different social groups regarding the population’s gender, age, and monthly household income, and it ensured that participants’ socio-demographic characteristics are representative of the characteristics of the surveyed communities. A total of 222 participants were recruited for the survey. By excluding responses with missing or incomplete data, the survey finally yielded valid data from 211 participants. 105 participants were recruited in SSP and 106 participants in TSW. As shown in Table 1, the participants cover a range of socio-demographic statuses through multiple axes, including gender, age, monthly household income, as well as education level and marital status. Please note that due to the difficulties in recruitment, we have slightly fewer people in the middle-aged group and the low-income group, but slightly more people in the middle-young age group and the middle-income group. Participants with respiratory conditions were rarely found in our sample (less than 3%), which matches well the actual situation. According to the rule of thumb, the sample size of participants with respiratory conditions is too small and cannot be considered adequate. Since the number of participants with respiratory conditions is small and not adequate for statistical analysis, the effects of pre-existing respiratory conditions are not considered in this study. Following this pilot study, our future data collection aims at a much larger sample size and an adequate number of participants who may report respiratory and other chronic health conditions, whereby we will discuss the effects of chronic health conditions on the associations between people’s perception of air quality and their health outcomes. Meanwhile, we did not receive any responses from other gender groups (e.g., transgenders) in Hong Kong, and we acknowledge that our data are not adequate for discussing the associations between people’s perceived air quality and their health outcomes for these non-binary gender groups (LGBTQ+).

### 2.3. Statistical Analysis

In this study, we first inspect the collected data through descriptive analyses. Percent stacked bar charts are used to visualize the disparities in participants’ perceived air quality and their self-reported frequency of respiratory symptoms with respect to different genders, age groups, household income levels, education levels, marital statuses, and geographic contexts. Mann–Whitney *U* tests are then used to examine the significance of differences between different groups along each socio-demographic axis and between different geographic contexts, respectively.

Spearman rank correlation coefficients [33] are employed to evaluate the association between people’s perceived air quality and their self-reported frequency of respiratory symptoms. Spearman rank correlation is a non-parameter correlation method for measuring the strength and degree of the association between two ordinal variables. The value of the Spearman rank correlation coefficient ($r_{s}$) ranges from −1 to 1. If $r_{s}$ > 0, the two variables rank in a similar order. On the contrary, if $r_{s}$ < 0, the two variables rank in the opposite order. The absolute value of $r_{s}$ indicates the degree of correlation. If $r_{s}$ is closer to ±1, the correlation between two ordinal variables is stronger.

Three levels of association are tested in this study. The first level considers the overall association among all investigated participants. Each variable of participants’ perceived air quality is paired with each variable of their self-reported frequency of respiratory symptoms to derive the Spearman rank correlation coefficient, respectively. The second level further considers different socio-demographic groups. The Spearman rank correlation coefficients of the same variable pairs as the first level are derived according to different genders, age groups, household income levels, education levels, and marital statuses, respectively. The third level considers the disparity in geographic contexts. The Spearman rank correlation coefficients of the same variable pairs as the first level are derived in SSP and TSW, respectively.

Finally, binary logistic regression models are employed to predict participants’ self-reported frequency of respiratory symptoms using their perceived air quality. Participants’ self-reported frequency of respiratory symptoms (cough, phlegm, and shortness of breath) are recoded into either low frequency (coded as 0) or high frequency (coded as 1). The original scores 1–3 are recoded as 0 and the original scores 4–6 are recoded as 1. We have developed two types of models. The first type (Type I model) includes only people’s perceived air quality to simulate the prediction that purely uses social media data and cannot concern socio-demographic characteristics. The second one (Type II model) further includes the adjustments of gender, age, monthly household income, education level, and marital status. In the Type II model, the variables of gender, age, monthly household income, education level, and marital status are reclassified as nominal and ordinal variables according to Table 1; these discrete variables are included as independent variables to control potential confounding effects of the socio-demographic characteristics. In both model types, we consider TSW as a reference to contrast the different geographic contexts between SSP and TSW. Nagelkerke R^2^, Cox & Snell R^2^, and −2 Log likelihood are used to indicate the robustness of the fitted models.

An adequate sample size is critical for a reliable estimation of correlation coefficients and significance tests. According to the rule of thumb, our sample size is adequate for descriptive analyses and multivariable regressions. Meanwhile, according to Schönbrodt and Perugini’s estimation [34], our sample size is adequate for the estimation of correlation coefficients along all the socio-demographic axes and in most sub-groups. 

## 3. Results

### 3.1. Descriptive Analyses of the Collected Data 

**Figure 2:**
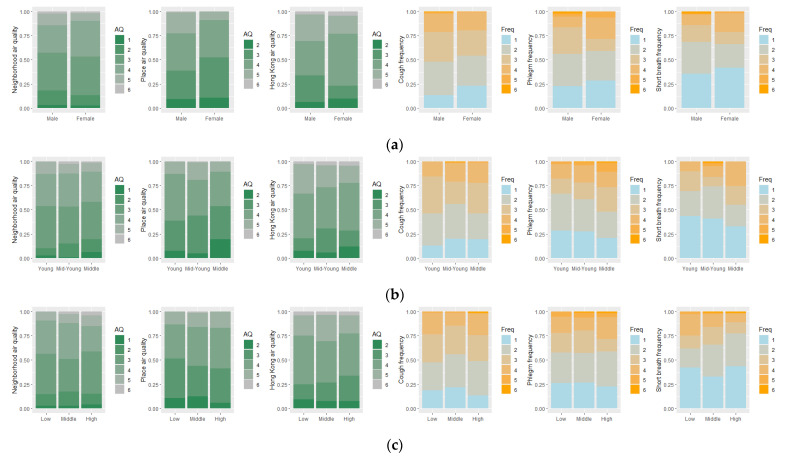

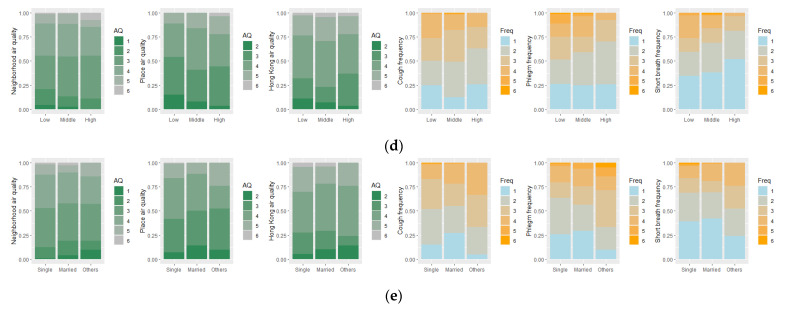
Distribution of participants’ perceived air quality and their self-reported respiratory symptoms regarding different socio-demographic axes: (**a**). gender, (**b**). age, (**c**). monthly household income, (**d**). education level, and (**e**). marital status. AQ is short for air quality.

**Figure 3:**
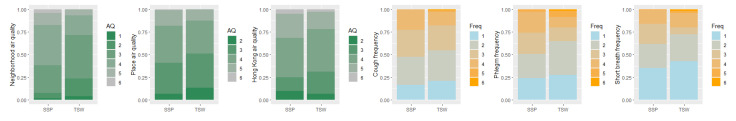
Distribution of participants’ perceived air quality and their self-reported respiratory symptoms in different geographic contexts. AQ is short for air quality.

As indicated by the collected data, there is no significant disparity in perceived air quality between different socio-demographic groups (Figure 2) and geographic contexts (Figure 3) except for a few notable variables. Regarding people’s perceived air quality, male participants reported worse perceived place air quality than female participants, middle-young participants reported worse perceived place air quality than middle-aged participants, and SSP participants reported much worse perceived neighborhood air quality than TSW participants (Figure 2a,b and Figure 3, and Table 2). Previous studies indicate that women tend to more frequently report worse air quality than men in the same situation [26,27,28]. However, other studies have indicated that women may have a smaller activity space than men [35,36,37], whereby we cannot assume that women and men have visited the same places with the same air quality conditions regarding their perceived place air quality. Our results suggest that men may visit places with worse air quality than women, and the disparity is statistically significant (Table 2) even though female participants may tend to report worse air quality. The difference between middle-young and middle-aged participants may have a similar reason, whereby our observations indicate that middle-young participants may visit places with worse air quality than middle-aged participants. To our best knowledge, the disparity in the perceived air quality between the participants of different age groups is a novel observation. This disparity may be due to the age-related differences in the sensitivity to air pollutants, daily mobility, and work and activity contexts, which needs further exploration. The difference between SSP and TSW is mainly due to the geographic contexts. SSP is the downtown area of Hong Kong, with heavier traffic and more crowded buildings, and thus may concentrate more air pollutants from vehicle emissions and lead to poorer air quality. In contrast, TSW is in a new town with a better design of open space, surrounded by villages and natural parks, and thus may be less affected by air pollution. The difference between other socio-demographic groups is not statistically different from 0, and we can assume equivalent observations for correlation analysis.

Regarding people’s self-reported frequency of respiratory symptoms, participants with lower education experience reported a higher frequency of shortness of breath than those with higher education experience; divorced and widowed participants reported a higher frequency of phlegm than single participants, and they also reported a higher frequency of cough than married participants (Figure 2d,e and Table 3). These observations indicate that disadvantaged socio-demographic groups such as those with lower education experience and being divorced or widowed may be more vulnerable to and suffering from respiratory symptoms. No significant disparity is observed between other socio-demographic groups as well as in different geographic contexts. Furthermore, we observed that 76% of participants being divorced or widowed are female, and 72% of participants with lower education experience are female, while no apparent disparities have been observed in other education level groups and marital status groups. These apparent gender disparities indicate essential socio-demographic inequalities between different genders in these disadvantaged groups.

### 3.2. Overall Associations between People’s Perceived Air Quality and Their Self-Reported Frequency of Respiratory Symptoms

The overall association between people’s perceived air quality and their self-reported frequency of respiratory symptoms is first examined for all participants without considering the disparities between different socio-demographic groups and in different geographic contexts. The results indicate a weak but significant association between people’s perceived overall Hong Kong air quality (Hong Kong air quality) and the frequency they had phlegm (Table 4). No significant association is observed using other indicators. This association is consistent with current studies that people’s perceived poor air quality can represent their actual exposure to poor air conditions [21,22,24]. However, since different groups of people may have distinct vulnerabilities to poor air conditions, the same exposure to poor air conditions may not yield similar respiratory symptoms. For example, older adults may have a greater susceptibility to airway inflammation than younger adults when they are exposed to a similar level of traffic-related air pollution [38]. The aggregated statistics of different groups of people may yield insignificant results and hinder the identification of population groups with higher health risks. This may be the reason why the results in Table 4 show only one weak association and many insignificant associations between people’s perceived air quality and their self-reported frequency of respiratory symptoms, while many studies showed a strong significant association between people’s perceived air quality and their exposure to poor air conditions. Consequently, it is necessary to examine the disparities in these associations for different groups of people to explore these more vulnerable groups.

### 3.3. Disparities in Associations between Different Socio-Demographic Groups

In this subsection, we investigate the associations between people’s perceived air quality and their self-reported frequency of respiratory symptoms for different socio-demographic groups. The most striking disparity is found between male participants and female participants (Table 5). Among the participants, male participants’ perceived air quality is significantly associated with their self-reported frequency of respiratory symptoms using multiple indicators employed in this study. In contrast, no significant association is observed for female participants. These results indicate that even though women may tend to report worse air quality than men, women’s perceptions of air quality may not necessarily be associated with their respiratory symptoms.

The disparities in associations have also been observed along other axes of the socio-demographic profiles (Table 6). For all three age groups, there are significant associations between their perceived air quality and their self-reported frequency of respiratory symptoms but in different symptom categories. The frequency for young participants having shortness of breath is significantly associated with their perceived place air quality, the frequency for middle-young participants having phlegm is significantly associated with their perceived Hong Kong air quality, and the frequency for middle-aged participants having a cough is significantly associated with their perceived place air quality. Disadvantaged groups with low and middle monthly household income and with low and middle education experience show significant associations using either perceived place air quality or Hong Kong air quality. Further, single participants show significant associations between perceived Hong Kong air quality and the frequency of both cough and phlegm. These results indicate that the associations between people’s perceived air quality and their self-reported frequency of respiratory symptoms are not general and solid, and these significant associations can be observed for only some socio-demographic groups and using specific indices. Furthermore, we observed that 68% of middle-aged participants have a lower education experience. The percentage of middle-aged participants in the low-education group is disproportionate to the socio-demographic profile of our dataset. However, the middle-aged group has a significant association between place air quality and cough frequency, while low education group has a significant association between place air quality and shortness of breath frequency. The inconsistent association indicates that age may not be a confounder of education level. Similarly, no potential confounding effect has been observed between any other pairs of variables from our dataset.

### 3.4. Disparities in Associations between Different Geographic Contexts

The associations between people’s perceived air quality and their self-reported frequency of respiratory symptoms are also examined with respect to different geographic contexts. Apparent disparities in the associations are observed in different geographic contexts between people’s perceived air quality and their self-reported frequency of respiratory symptoms (Table 7). SSP shows multiple significant associations regarding all sorts of self-reported respiratory symptoms. In contrast, no significant association is observed in TSW. SSP belongs to the downtown region of Hong Kong that faces more severe air quality issues. It is easier for air pollutants to reach higher concentration levels in the crowded neighborhoods in SSP (Figure 1c), whereby people’s perceived air quality is more strongly correlated to their self-reported frequency of respiratory symptoms. In contrast, TSW is better planned and closer to rural areas (Figure 1b), where air pollutants are less easily concentrated. That may be the reason why no significant association is observed in TSW.

Another thing to notice is that there is no significant association between people’s perceived neighborhood air quality and their self-reported frequency of respiratory symptoms either in SSP or TSW (Table 7). This phenomenon is also very common regarding the overall association (Table 4) and considering different socio-demographic groups (Table 5 and Table 6). This phenomenon can be attributed to participants’ complex real-time contexts, including being indoors and outdoors, adjacent to streets or not, and some other confounding factors. For example, participants may be exposed to indoor air pollutants while cooking for a short period. This short-term but strong influencing factor may bias the long-term overall perception of air quality, leading to a recall bias, weakening the association between participants’ long-term perception of air quality and their chronic respiratory symptoms. We also collected objective air quality measurements from the air quality monitoring stations in Hong Kong via the Environmental Protection Interactive Centre (https://cd.epic.epd.gov.hk/EPICDI/air/station/, accessed on 10 November 2022). The data collected from Yuen Long station is compared against participants’ perceived neighborhood air quality in TSW, and the data collected from Sham Shui Po station is compared against participants’ perceived neighborhood air quality in SSP. The mean and median values of air pollutants (e.g., PM_2.5_, PM_10_, NO_2_, NO_x_, O_3_, and SO_2_) in the past six months from the moment when participants took the survey do not show any significant correlation with participants’ perceived neighborhood air quality, regardless of the correlation coefficient estimations using Pearson’s, Kendall’s, or Spearman’s methods. These results further confirm that people’s perception of their neighborhood’s ambient air quality cannot be used as a robust indicator to link their perceived air quality with their respiratory symptoms.

### 3.5. Predicting the Frequency of Respiratory Symptoms Using People’s Perceived Air Quality

In the last subsection, we attempt to predict participants’ self-reported frequency of respiratory symptoms using their perceived air quality. The results in Table 8 indicate that for the entire sample of participants, it is impossible to use participants’ perceived air quality to predict their self-reported frequency of respiratory symptoms, even when controlling for gender, age, household income, education level, and marital status (Type II). By controlling the effects of participants’ socio-demographic statuses, the logistic regression models show better agreement between observations and predictions. On the other hand, some socio-demographic groups do have significantly different effect sizes than other groups, such as males, young participants, and disadvantaged groups with low and middle monthly household income and with low and middle education experience. These results further indicate that there are associations between people’s perceived air quality and their self-reported frequency of respiratory symptoms for only some specific socio-demographic groups rather than the entire population.

## 4. Discussion

### 4.1. Interpretation of the Study Results

This study is the first attempt to examine the disparities in the associations between people’s perceived air quality and their self-reported respiratory symptoms along different socio-demographic axes and in different geographic contexts. Beyond past studies that have only discussed the associations between people’s perceived air quality and their exposure to air pollution, this study extends the connection to the concrete health burden of poor air conditions such as respiratory symptoms, and then systematically discusses the disparities in such associations between different socio-demographic groups and different geographic contexts. We observed inconsistent associations between different genders, age groups, household income levels, education levels, marital statuses, and geographic contexts. Significant associations between perceived air quality and self-reported frequency of respiratory symptoms are observed for men, people in all age groups, those with low and middle household income and education level, those who are single, and those living in the old town. In contrast, insignificant associations are observed for all other socio-demographic groups.

The most striking disparity we observed is that between different genders. Past studies have shown that women tend to more frequently report worse air quality than men when they are exposed to a similar level of poor air quality [26,27,28]. However, our results indicate that women’s perceptions of air quality may not necessarily be linked to the actual health burden of poor air quality on women’s respiratory systems. Women may be more sensitive to air quality issues and more frequently report poor air quality before the poor ambient air conditions trigger and worsen women’s respiratory symptoms. Men may be less sensitive to the change in ambient air quality. Thus, when men perceive poor air quality, it may indicate that their ambient air quality is bad enough to trigger or worsen their respiratory symptoms. That may be the reason why for male participants, there are more significant associations between their perceived air quality and their self-reported frequency of respiratory symptoms while there is no such association for female participants.

We also observe disparities between different geographic contexts. This is a typical manifestation of spatial non-stationarity [39]. The places with better-designed facilities and more green space may less likely to concentrate air pollutants. The concentration of air pollutants in these places may not trigger severe respiratory burdens and people’s perception of air quality in these places may be more affected by personal attributes. In our study, TSW is a typical community of this sort and we do not observe any significant association. In contrast, significant associations may be observed in places that more easily concentrate air pollutants, such as SSP. Moreover, we find that people’s perception of neighborhood air quality is not a solid indicator associated with their respiratory burden. This phenomenon can be understood through the uncertain geographic context problem (UGCoP) [40]. As a manifestation of the UGCoP, it is difficult to accurately identify the precise contextual areas for people’s perceived air quality, and it is also difficult for people to remember the accurate timing and duration when they perceived air conditions. As a result, inaccurate perceptions of air quality may include recall biases and lead to their insignificant associations with people’s respiratory burden.

In summary, we find that people’s perceptions of air quality are only significantly associated with their respiratory burden for particular socio-demographic groups, in specific geographic contexts, and using specific indicators. These associations may not exist for the entire population.

### 4.2. Implications for Environmental Justice Studies

Environmental justice is drawing increasing global attention and pertinent research is adapting to the age of big data [41]. The concept of environmental justice first emerged in the 1980s in the United States after a series of anti-toxicity and other social movements. It has been found that the heaviest toxic burdens of industrial pollution are concentrated on disadvantaged groups (e.g., the black and Hispanic ethnic minorities) and those of lower socioeconomic statuses [42,43,44]. An important aim of these environmental justice movements is the visualization of the uneven distribution of exposure to adverse environmental factors (i.e., environmental injustice). Efforts are then made to challenge and overcome these injustices. This framework is then gradually adopted in other places and regions of the world [45]. Environmental justice requires sufficient data to uncover structural injustices, which in turn has encouraged relevant studies embracing big data in recent decades. The big data phenomenon has led to the emergence of a large volume of data from various sources (e.g., citizen sensing [46], crowdsourced data [47], and social media data [48]). Social media data is one of the big data sources that can be applied to environmental justice assessment. This big data source currently is under intensive discussion since it has several advantages, including broad spatial coverage and high spatiotemporal resolutions [21], high flexibility to cover diverse health concerns [22], fast response to extreme events without presetting [23], and no requirement of extra devices.

Social media data (e.g., data collected from Twitter and Sina Weibo) have shown high correspondence with momentary exposure to negative health impact factors such as heavy air pollution [22]. The high correspondence shows that people’s perceptions and linguistic expressions of poor air conditions can be employed to indicate their exposure to poor air quality. However, our results indicate that researchers need to be careful when using social media data to represent and visualize structural differences and environmental injustice. This is because disparities exist in the associations between people’s perceived air quality and their health outcomes, such as respiratory symptoms, for different socio-demographic groups and in different geographic contexts. For example, apparent disparities exist between men and women and between participants living in old towns and new towns. As a result, the perceptions and linguistic expressions of ambient air quality obtained via social media sources may not represent the true inequality in environmental health burdens (e.g., the effects of air pollution on people’s respiratory systems), given the inconsistent associations in different socio-demographic groups and geographic contexts.

### 4.3. Limitations of This Study

This study has some limitations. First, it is a cross-sectional study and thus cannot examine the temporal variation of the associations between people’s perceived air quality and their self-reported frequency of respiratory symptoms. Second, the sample size of this study is adequate but small. As a result, some of the socio-demographic groups surveyed in the study cannot provide very reliable associations even though we can observe weak but significant associations (e.g., young participants). Third, we only considered the confounding effects of socio-demographic characteristics. Other factors, including chronic health conditions (e.g., respiratory conditions, cardiovascular conditions, and diabetes) and individual lifestyles (e.g., cigarette smoking, alcohol drinking, and healthcare), may also confound the effects of the variables we used in this pilot study. However, since relevant valid responses are rare in the stratified sampling, we did not receive adequate responses from participants and thus our data collection is not adequate to discuss these potential confounding effects. Future research will benefit from using a longitudinal design that further analyzes the associations between people’s perceived air quality and health outcomes such as respiratory symptoms and uses larger sample sizes.

## 5. Conclusions

In this study, we examined the associations between people’s perceived air quality and their self-reported frequency of respiratory symptoms. Apparent disparities are observed in the associations between different socio-demographic groups and different geographic contexts. The most striking disparities are between genders and geographic contexts. There are significant associations between perceived air quality and self-reported frequency of respiratory symptoms for the male participants and people living in the old town, while insignificant associations are observed for the female participants and those living in the new town. We also found that the associations only exist for particular socio-demographic groups, in specific geographic contexts, and using specific indicators, rather than the entire population. Our results have important implications for environmental health studies that concern people’s perceptions of local environments, especially those using social media data. Due to the inconsistent associations between perceived environments such as ambient air quality and health outcomes such as respiratory symptoms for different groups of people and in different geographic contexts, researchers need to scrutinize these perception data before addressing the public’s concerns regarding local environments.

## Figures and Tables

**Table 1:** The socio-demographic profile of investigated participants.

Socio-Demographic Characteristics		SSP (Old Town)		TSW (New Town)		Both	
		Sample	Census Statistics	Sample	Census Statistics	Sample	Census Statistics
Gender	Male	47 (44.8%)	46%	51 (48.1%)	47%	98 (46.4%)	47%
	Female	58 (55.2%)	54%	55 (51.9%)	53%	113 (53.6%)	53%
Age	Young 18–24	17 (16.2%)	14%	22 (20.8%)	16%	39 (18.5%)	15%
	Mid-young 25–44	51 (48.6%)	42%	54 (50.9%)	39%	105 (49.8%)	40%
	Middle 45–64	37 (35.2%)	44%	30 (28.3%)	46%	67 (31.8%)	45%
Monthly household Income ^1^	Low	47 (44.8%)	55%	29 (27.4%)	45%	76 (36.0%)	50%
	Middle	34 (32.4%)	27%	48 (45.3%)	34%	82 (38.9%)	30%
	High	24 (22.9%)	18%	29 (27.4%)	21%	53 (25.1%)	20%
Chronic health conditions	Respiratory	2 (1.9%)	-	4 (3.8%)	-	6 (2.8%)	-
	Others	17 (16.2%)	-	14 (13.2%)	-	31 (14.7%)	-
	None	86 (81.9%)	-	88 (83.0%)	-	174 (82.5%)	-
Education level ^2^	Low	37 (35.2%)	-	35 (33.0%)	-	72 (34.1%)	-
	Middle	55 (52.4%)	-	57 (53.8%)	-	112 (53.1%)	-
	High	13 (12.4%)	-	14 (13.2%)	-	27 (12.8%)	-
Marital status	Single	53 (50.5%)	-	59 (55.7%)	-	112 (53.1%)	-
	Married	41 (39.0%)	-	37 (34.9%)	-	78 (37.0%)	-
	Others ^3^	11 (10.5%)	-	10 (9.4%)	-	21 (10.0%)	-
Total		105 (100%)	100%	106 (100%)	100%	211 (100%)	100%

^1^ Monthly household income: the low-income group has an income of less than 20,000 Hong Kong dollars (HKD), the middle-income group has an income of 20,000~39,999 HKD, and the high-income group has an income of 40,000 HKD or above. ^2^ Education level: the low group graduated from middle school or lower, the middle group had a bachelor’s degree or certification, and the high group had a master’s degree or higher. ^3^ Other marital statuses include divorced and widowed.

**Table 2:** Mann–Whitney *U* tests of difference in participants’ perceived air quality regarding different socio-demographic axes and geographic contexts.

Axis	Paired Groups		Neighborhood Air Quality		Place Air Quality		Hong Kong Air Quality	
			*U*	*p*-Value	*U*	*p*-Value	*U*	*p*-Value
Gender	Male	Female	5334	0.630	6542 *	**0.016**	5474	0.881
Age	Young	Mid-young	2085	0.860	2030	0.937	2256	0.322
	Young	Middle	1428	0.403	1543	0.102	1480	0.225
	Mid-young	Middle	3781	0.385	4182 *	**0.027**	3626	0.718
Household income	Low	Middle	2984	0.629	2920	0.473	3020	0.722
	Low	High	1995	0.925	1776	0.225	2159	0.460
	Middle	High	2236	0.768	2064	0.604	2388	0.306
Education level	Low	Middle	3829	0.545	3394	0.055	3581	0.174
	Low	High	893	0.518	788	0.127	988	0.898
	Middle	High	1468	0.806	1460	0.771	1715	0.251
Marital status	Single	Married	4741	0.292	4884	0.142	4740	0.291
	Single	Others	1261	0.582	1232	0.719	1244	0.659
	Married	Others	813	0.961	764	0.626	793	0.814
Community	SSP	TSW	7678 **	**<0.001**	6330	0.068	6150	0.162

* True location shift is significantly not equal to 0 at the 0.05 level. ** True location shift is significantly not equal to 0 at the 0.01 level. All significant values are highlighted in bold font.

**Table 3:** Mann–Whitney *U* tests of difference in participants’ self-reported frequency of respiratory symptoms regarding different socio-demographic axes and geographic contexts.

Axis	Paired Groups		Cough		Phlegm		Short Breath	
			*U*	*p*-Value	*U*	*p*-Value	*U*	*p*-Value
Gender	Male	Female	6082	0.202	5566	0.946	5606	0.870
Age	Young	Mid-young	2196	0.490	1946	0.639	2010	0.862
	Young	Middle	1295	0.940	1055	0.090	1064	0.099
	Mid-young	Middle	3282	0.445	3028	0.113	2978	0.076
Household income	Low	Middle	3471	0.201	3141	0.930	3082	0.903
	Low	High	1972	0.837	1930	0.678	2234	0.269
	Middle	High	1875	0.163	2066	0.618	2484	0.142
Education level	Low	Middle	3925	0.754	4225	0.574	4417	0.255
	Low	High	1096	0.316	1150	0.149	1244 *	**0.026**
	Middle	High	1785	0.129	1720	0.251	1800	0.107
Marital status	Single	Married	4639	0.452	4226	0.694	4447	0.825
	Single	Others	880	0.056	807 *	**0.018**	946	0.140
	Married	Others	588 *	**0.042**	608	0.065	652	0.136
Community	SSP	TSW	6034	0.272	6145	0.177	6002	0.302

* True location shift is significantly not equal to 0 at the 0.05 level. All significant values are highlighted in bold font.

**Table 4:** The Spearman rank correlation coefficients of the total sample.

(N = 211)	Neighborhood Air Quality		Place Air Quality		Hong Kong Air Quality	
	$r_{s}$	*p*-Value	$r_{s}$	*p*-Value	$r_{s}$	*p*-Value
Cough	0.002	0.486	0.086	0.106	0.113	0.051
Phlegm	−0.007	0.461	0.086	0.107	0.154 *	**0.013**
Short breath	0.075	0.139	0.096	0.082	0.091	0.094

* Correlation is significant at the 0.05 level (1-tailed). The significant value is highlighted in bold font.

**Table 5:** The Spearman rank correlation coefficients in the categories of gender.

	Category	Neighborhood Air Quality		Place Air Quality		Hong Kong Air Quality	
		$r_{s}$	*p*-Value	$r_{s}$	*p*-Value	$r_{s}$	*p*-Value
Male (N = 98)	Cough	0.027	0.396	0.120	0.119	0.190 *	**0.031**
	Phlegm	0.112	0.137	0.205 *	**0.021**	0.198 *	**0.026**
	Short breath	0.195 *	**0.027**	0.169 *	**0.049**	0.190 *	**0.031**
Female (N = 113)	Cough	−0.010	0.460	0.033	0.365	0.055	0.282
	Phlegm	−0.110	0.122	−0.017	0.431	0.122	0.099
	Short breath	−0.029	0.381	0.040	0.336	0.002	0.490

* Correlation is significant at the 0.05 level (1-tailed). All significant values are highlighted in bold font.

**Table 6:** The significant Spearman rank correlation coefficients in the categories of age, monthly household income, education level, and marital status ^1^.

Socio-Demographic Characteristics		N	Perceived Air Quality	Respiratory Symptom	*r_s_*	*p*-Value ^2^
Axis	Group					
Age	Young	39	Place air quality	Short breath	0.290	0.037
	Middle-Young	105	HK air quality	Phlegm	0.254	0.004
	Middle	67	Place air quality	Cough	0.244	0.023
Household income	Low	76	Place air quality	Short breath	0.209	0.035
	Middle	82	HK air quality	Cough	0.188	0.046
Education level	Low	72	Place air quality	Short breath	0.211	0.038
	Middle	112	HK air quality	Phlegm	0.170	0.037
Marital status	Single	112	HK air quality	Cough	0.190	0.022
	Single	112	HK air quality	Phlegm	0.217	0.011

^1^ Insignificant correlation coefficients in other groups and using other indices are not reported in the table. ^2^ 1-tailed tests.

**Table 7:** The Spearman rank correlation coefficients in SSP and TSW.

	Category	Neighborhood Air Quality		Place Air Quality		Hong Kong Air Quality	
		$r_{s}$	*p*-Value	$r_{s}$	*p*-Value	$r_{s}$	*p*-Value
SSP (N = 105)	Cough	−0.006	0.474	0.183 *	**0.031**	0.202 *	**0.019**
	Phlegm	0.002	0.494	0.217 *	**0.013**	0.270 **	**0.003**
	Short breath	0.096	0.165	0.164 *	**0.048**	0.126	0.100
TSW (N = 106)	Cough	−0.065	0.253	−0.024	0.402	−0.003	0.488
	Phlegm	−0.084	0.196	−0.066	0.251	−0.004	0.485
	Short breath	−0.007	0.470	0.012	0.451	0.023	0.408

* Correlation is significant at the 0.05 level (1-tailed). ** Correlation is significant at the 0.01 level (1-tailed). All significant values are highlighted in bold font.

**Table 8:** Results of the binary logistic regression models for predicting participants’ self-reported frequency of respiratory symptoms in SSP and TSW, N = 211.

Variables		Cough		Phlegm		Short Breath	
		Type I	Type II	Type I	Type II	Type I	Type II
Constant		−2.143 **	−2.221 *	−2.093 **	−2.757 **	−2.435 **	−4.533 **
Neighborhood air quality		−0.198	−0.159	−0.172	−0.215	−0.048	−0.039
Place air quality		0.174	0.105	−0.003	0.148	−0.076	0.105
Hong Kong air quality		0.157	0.238	0.313	0.300	0.368	0.330
Geographic context	SSP	0.373	0.347	0.402	0.388	−0.262	−0.557
	TSW	Reference					
Gender	Male		0.388		−0.765 **		−0.325
	Female	Reference					
Age	Young		0.084		−0.652		−1.718 **
	Middle-Young		0.375		−0.340		−0.631
	Middle	Reference					
Monthly household income	Low		−0.546		−0.870 *		0.681
	Middle		−0.898 *		−0.779 *		0.074
	High	Reference					
Education level	Low		1.151		1.649 *		2.030 *
	Middle		0.351		1.752 **		1.703
	High	Reference					
Marital status	Single		−0.684		0.023		0.737
	Married		−0.660		−0.118		0.031
	Others	Reference					
Nagelkerke R^2^		0.019	0.084	0.028	0.125	0.026	0.146
Cox & Snell R^2^		0.012	0.054	0.019	0.082	0.016	0.089
−2 Log likelihood		210.746	201.760	222.296	208.147	195.644	179.339

* Logit is significantly different from 0 at the 0.10 level. ** Logit is significantly different from 0 at the 0.05 level. All significant values are highlighted in bold font.

## Data Availability

Data sharing is not applicable to this article.
